# Long-Term Non-Cancer Risks in People with *BRCA* Mutations following Risk-Reducing Bilateral Salpingo-Oophorectomy and the Role of Hormone Replacement Therapy: A Review

**DOI:** 10.3390/cancers15030711

**Published:** 2023-01-24

**Authors:** Amanda S. Nitschke, Helena Abreu do Valle, Lesa Dawson, Janice S. Kwon, Gillian E. Hanley

**Affiliations:** Department of Obstetrics and Gynaecology, University of British Columbia, Vancouver, BC V6Z 2K8, Canada

**Keywords:** *BRCA*, hereditary breast and ovarian cancer syndrome, risk-reducing salpingo-oophorectomy, hormone replacement therapy

## Abstract

**Simple Summary:**

People with *BRCA* mutations are at high risk for ovarian and breast cancer. In order to greatly reduce their risks of these cancers, people with *BRCA* mutations undergo surgery between the ages of 35–45 to remove both ovaries and fallopian tubes. However, this type of surgery results in early menopause, which leads to negative long-term health effects. Little is known about these health effects in people with *BRCA* mutations. In addition, uncertainty surrounds the safety and effectiveness of hormone replacement therapy to treat these health effects and improve quality of life. This review summarizes the current research on the long-term health consequences of early surgical menopause in people with *BRCA* mutations and highlights the existing research in support of hormone replacement therapy use in this population.

**Abstract:**

Risk-reducing bilateral salpingo-oophorectomy (RRBSO) is the gold standard preventative option for *BRCA* mutation carriers at high risk for ovarian and breast cancer. However, when performed at the recommended ages of 35–45 years, RRBSO induces immediate premature surgical menopause, along with the accompanying adverse psychosocial, cardiovascular, bone, and cognitive health consequences. While these health consequences have been thoroughly studied in the general population, little is known about the long-term health outcomes in the *BRCA* population. Hormone replacement therapy (HRT) until the average age of natural menopause can help mitigate these health risks, yet the initiation of HRT is a complex decision among *BRCA* carriers due to concern of increasing the already high risk of breast cancer in these people. This review summarizes the current research on long-term non-cancer risks in *BRCA* carriers following RRBSO-induced premature surgical menopause, and highlights the existing evidence in support of HRT use in this population.

## 1. Introduction

Breast cancer and ovarian cancer are the first and eighth leading causes of cancer death among women worldwide [1]. Hereditary and genetic predisposition play an important role in an individual’s susceptibility to these cancers [2]. In particular, germline mutations in genes responsible for DNA repair, such as breast cancer susceptibility genes 1 and 2 (*BRCA1* and *BRCA2*), are found to greatly increase an individual’s risk [3]. While the lifetime risk of breast cancer is 12% in the general population [4], the cumulative risk by the age of 80 increases to 72% among *BRCA1* mutation carriers and 69% among *BRCA2* mutation carriers [5]; and with regard to ovarian cancer, the corresponding risk estimates are 1.3% in the general population [4], increasing to 44% and 17% among *BRCA1* and *BRCA2* carriers, respectively [5].

In order to manage this increased cancer risk, people with *BRCA* mutations are provided with preventative options, including chemoprevention for breast cancer (e.g., tamoxifen) and ovarian cancer (e.g., oral contraceptives), and surgical options, such as risk-reducing mastectomy and risk-reducing salpingo-oophorectomy (RRBSO) [6,7]. Currently, no effective screening method for ovarian cancer exists. The most recent evidence from the UK Collaborative Trial of Ovarian Cancer Screening revealed that after a median of 16.3 years of follow-up among 202,562 randomized participants, there was no statistically significant reduction in ovarian cancer or tubal cancer deaths in the screening groups [8]. Therefore, in *BRCA* carriers, RRBSO is the primary recommendation [9,10]. RRBSO involves the surgical removal of both ovaries, as well as both fallopian tubes, as fallopian tubes are increasingly being recognized as the site of origin for the most common and lethal form of ovarian cancer, high-grade serous ovarian cancer [11,12]. Following this surgery, the risks of breast and ovarian cancers in *BRCA* carriers are reduced by 46–75% and 80–96%, respectively, with overall low surgical morbidity [13,14,15,16,17]. As such, guidelines from the National Comprehensive Cancer Network (NCCN) recommend people with *BRCA1* mutations to undergo RRBSO between the ages of 35 to 40 and upon completion of childbearing. Since the onset of ovarian cancer among *BRCA2* mutation carriers is approximately 8 to 10 years later than in *BRCA1* mutation carriers, the recommended timing of RRBSO is delayed to ages 40 to 45 [18].

Despite the effectiveness of undergoing RRBSO at the recommended age, the removal of the ovaries is not recommended for the general population, as it places people into immediate surgical menopause with an abrupt and significant decline in both estrogen, progesterone, and androgen levels. As a result, surgical menopause is associated with various adverse physical, mental, and cognitive health outcomes [19]. In contrast, natural menopause, which occurs at an average age of 51, is accompanied by a gradual decline in sex hormone levels [20]. In addition, the ovaries continue to produce considerable amounts of testosterone and androstenedione for many years after the natural menopausal transition, and these androgens are then converted to estrogen peripherally [21].

While research has thoroughly investigated the health outcomes of premature surgical menopause in the general population, relatively little is known about the long-term health consequences in high-risk people with *BRCA* mutations [22]. In addition, while hormone replacement therapy (HRT) is readily recommended and used to mitigate many of these health concerns in the general population, its use among *BRCA* carriers is a more complex decision due to the concern of increasing the already high risk of breast cancer in these people [23,24,25]. The aim of this review is to summarize the current research on long-term non-cancer risks in *BRCA* carriers following RRBSO-induced premature surgical menopause and highlight the existing evidence in support of HRT use in this population.

## 2. Adverse Outcomes following Premature Surgical Menopause in The General Population

Most of the research to date regarding the long-term consequences of premature surgical menopause are from data generated in the general population, and not among those with a *BRCA* mutation. In the general population, research has clearly shown that premature surgical menopause is associated with reductions in quality of life, cardiovascular health, bone health, cognitive health, and an overall increased risk of mortality [26].

Premature surgical menopause is reported to increase vasomotor symptoms, reduce sleep quality [27], and increase sexual dysfunction, such as experiencing a loss of libido, dyspareunia, genital atrophy, and genitourinary syndrome [28,29,30,31]. More recent data have also suggested an increased risk for depression and anxiety following premature surgical menopause [32]. Studies have also illustrated important increased risks for cardiovascular disease (CVD). A meta-analysis recently reported a pooled estimate for the relative risk of CVD among women who underwent bilateral oophorectomy (BO) compared to premenopausal women of 2.62 (95% CI 2.05, 3.35). Further, when looking at BO before the age of 50 years compared to after, the pooled relative risk estimate substantially increased to 4.55 (95% CI 2.56, 8.01) [33]. The Mayo Clinic Cohort Study of Oophorectomy and Aging [34], a cohort from the Swedish Health Care Registers [35], and the Nurses’ Health Study (NHS) [36] have similarly found an increased risk of CVD associated with premenopausal BO.

Bone loss is also a concern following premature surgical menopause. Following natural menopause, small quantities of estrogen and androgen (the latter of which is then converted to estrogen peripherally) are still released, which is understood to provide some protection against osteoporosis compared to women who underwent BO-induced surgical menopause [37]. One study found that when BO was performed on women under the age of 45 years, the risk of bone fracture was 3.64-fold (95% CI 1.01, 13.04) compared to women who had the surgery after the age of 45 [38].

There is also growing evidence suggesting that estrogen may have neuroprotective properties, and thus, an abrupt loss to this exposure following BO may increase the risk of cognitive decline and neurodegenerative disease [39]. A 2019 meta-analysis suggested that BO at less than 45 years of age was associated with an increased risk of dementia (aHR 1.70 [95% CI 1.07, 1.43]) and a faster global cognitive decline [40]. Further, a 2022 study reported that premenopausal BO before the age of 43 was associated with an increased risk of Parkinson’s disease (HR 5.00 [95% CI 1.10, 22.70]) and parkinsonism (HR 7.67 [95% CI 1.77, 33.27]) [41].

Total mortality from all causes is also consistently reported to be higher among women who underwent premenopausal BO [41,42,43,44,45]. A 2021 population-based study following 200,549 women for 12 years reported that, compared with ovarian conservation, BO increased rates of all cause mortality in women of less than 45 years (HR 1.31 [95% CI 1.18, 1.45]) and 45-49 years of age (HR 1.16 [95% CI 1.04, 1.30]) [46].

## 3. Hormone Replacement Therapy following Premenopausal Bilateral Oophorectomy

### 3.1. General Population

Following premenopausal BO, hormone replacement therapy (HRT) is indicated until the age of the expected natural menopause for women without a personal history of breast cancer [47]. HRT is recommended as a preventative treatment for bone mineral density (BMD) loss and fractures in postmenopausal women [48]. Further, HRT is shown to reduce the risk for CVD among those undergoing premature surgical menopause [33,35]. Estrogen therapy is also reported to decrease the risk of all cause mortality by 40% in women who had BO before the age of 45 [45].

Despite the reported efficacy of HRT in reducing morbidity and mortality associated with menopause, results from the 2002 Women’s Health Initiative (WHI) study led to a significant decline in HRT use overall and has had a lasting effect on perceptions of HRT safety. This was particularly a result of its finding that combined estrogen and progestogen therapy (EPT) increased breast cancer risk [49]. Since then, growing evidence suggests that the type of progesterone may be differentially associated with breast cancer risk [50,51]. A recent population-based study reported that EPT with synthetic progestins was associated with increased odds of breast cancer (OR 1.28 [95% CI 1.22, 1.35]), while EPT with micronized, or bioidentical, progestogens was not associated with breast cancer (OR 0.99 [95% CI 0.55, 1.79]) [52]. Further, a systematic review of micronized progesterone EPT and its impact on breast cancer risk concluded that this formulation of HRT does not increase breast cancer risk for up to 5 years of treatment, and that longer durations of use are likely not associated with breast cancer risk as well [53].

Nevertheless, the WHI results have almost certainly influenced the low HRT uptake in premature surgical menopause patients, including in a Canadian study by Jang et al. [54]. They found that between 2004 and 2014, only 55.3% of women had ever used HRT, with a statistically nonsignificant decline in HRT users throughout the 10-year study period. Further, among HRT users, almost 50% had a prescription history of less than 1 year [54]. Together, these findings highlight that HRT initiation is too low and sustained use in not long enough.

### 3.2. BRCA Mutation Carriers

While there are noted challenges with HRT use following premature surgical menopause in the general population, there is also complexity for the *BRCA* mutation population driven by the concern that use may counteract the breast cancer risk reduction of undergoing RRBSO. There are no randomized clinical trials examining HRT use in *BRCA* mutation carriers following RRBSO [19]. Without this evidence, along with the negative public perceptions of HRT safety, many clinicians and patients remain hesitant to use HRT [19]. This hesitation may be heightened among *BRCA1* and *BRCA2* mutation carriers due to their differing baseline risks of breast cancer. Women with *BRCA1* mutations are commonly hormone receptor-negative, while women with *BRCA2* mutations are usually estrogen and progesterone reception-positive [55]. This leads to uncertainty on whether HRT management may influence breast cancer risk differently for these two high-risk populations [19].

Overall, reported rates of HRT use in the *BRCA* population are low, with studies reporting an uptake of between 8 to 47% following premenopausal RRBSO [56,57,58,59,60,61]. Canadian studies found that while the average age of RRBSO was approximately 40 years, only 44 to 47% of these women received HRT [58,59]. Further, the average duration of treatment was only 3.5 years. Another study found that among the 20% of *BRCA* mutation carriers who initiated HRT following RRBSO, the average length of treatment was only 2.8 years [57]. Considering the many adverse health consequences of premature surgical menopause, and the success HRT has at minimising these health risks, HRT use among *BRCA* carriers should ideally be sustained until closer to the average age of natural menopause, if sufficient research supports its safety.

## 4. HRT Use and Breast Cancer Risk in *BRCA* Mutation Carriers

To date, eight observational studies have evaluated the effect of HRT on breast cancer risk exclusively in *BRCA* mutation carriers (Table 1). The Prevention and Observation of Surgical Endpoints (PROSE) prospective cohort study followed 462 *BRCA* mutation carriers for an average of 3.6 years to investigate breast cancer risk depending on HRT use [62]. In this cohort, 155 women underwent RRBSO at an average age of 42.7, and 93 received HRT. They reported that RRBSO was significantly associated with a reduced risk of breast cancer (HR 0.40 [95% CI 0.18, 0.92]) and this risk reduction was not influenced by the use of HRT (HR 0.37 [95% CI 0.14, 0.96]) [62]. Different formulations of HRT were examined, with 50 women receiving estrogen therapy (ET) and 34 receiving EPT; no difference in breast cancer risk was observed [62]. Six years later, Domchek et al. [63] published an extension and follow-up to the 2005 study from Rebbeck et al. [62]. This prospective cohort study included 1299 *BRCA* mutation carriers, of which 321 women underwent RRBSO and were subsequently followed for an average of 5.4 years. Post-RRBSO, 45% of women received HRT. Results were in agreement with Rebbeck et al., showing that HRT use following RRBSO was not associated with an increase in risk of breast cancer in *BRCA1* and *BRCA2* mutation carriers (HR 0.52 [95% CI 0.30, 0.92] and HR 0.24 [95% CI 0.05, 1.03], respectively). Further, in *BRCA1* mutation carriers, HRT use without RRBSO was also shown to be associated with a reduced risk of breast cancer (HR 0.29 [95% CI 0.13, 0.69]). Breast cancer risk was not increased for both ET and EPT [63].

Eisen et al. [63] conducted a retrospective matched case-control study, including 236 case-control pairs of *BRCA1* mutation carriers. The average duration of HRT use was 3.7 years for cases and 4 years for controls. Compared to women who never used HRT, HRT use was associated with a reduction in breast cancer risk (OR 0.58 [95% CI 0.35, 0.96]). In addition, upon investigating the impact of different formulations of HRT, authors reported a statistically significant reduction in breast cancer risk associated with ET (OR 0.51 [95% CI 0.27, 0.98]) and a statistically nonsignificant risk reduction associated with EPT (OR 0.66 [95% CI 0.34, 1.27]) [64].

An extension of this study was later conducted by Kotsopoulos et al. [65] who reported on 432 match case-control pairs with *BRCA1* mutations. Compared to Eisen et al. [64], this study found no association between the risk of breast cancer and the use of HRT (OR 0.80 [95% CI 0.55, 1.16]). The average duration of HRT was 4.42 years and 4.27 years for cases and controls, respectively. ET and EPT were both not associated with increased or decreased odds of breast cancer. Of note, the majority of women in this study, 327 (75.7%), experienced natural menopause. No significant difference was observed when women who experienced natural versus premature surgical menopause were examined separately (surgical menopause: OR 1.06 [95% CI 0.58, 1.96] vs. natural menopause: OR 0.72 [95% CI 0.44, 1.18]) [65].

The same research group published a prospective cohort study in 2018, including 872 *BRCA1* mutation carriers undergoing RRBSO, a subset of which overlapped with the previous case-control study [66]. They found that HRT use following RRBSO was not associated with an increased risk of breast cancer (HR 0.97 [95% CI, 0.62, 1.52]). However, after a 10-year follow-up period, they observed a significantly lower breast cancer risk among *BRCA1* mutation carriers who used ET compared to EPT (12% vs 22%, *p* = 0.04) [66]. More specifically, each year of ET use led to an observed 8% reduction in breast cancer risk (HR 0.92 [95% CI 0.83, 1.01]), in contrast to EPT use, where each year led to a nonsignificant 8% increase in risk (HR 1.08 [95% CI 0.92, 1.27]). These associations were more pronounced for women who underwent RRBSO before the age of 45, with each year of ET associated with a 18% reduction in breast cancer risk (HR 0.82 [95% CI 0.69, 0.97]), and each year of EPT associated with a nonsignificant 14% increase in breast cancer risk (HR 1.14 [95% CI 0.90, 1.46]). Overall, authors concluded that ET after oophorectomy in *BRCA1* carriers does not increase the risk of breast cancer: however, the possible adverse effect of EPT use in this population requires further investigation [66].

More recently, Michaelson-Cohen et al. [67] conducted a retrospective cohort study including 306 *BRCA1/2* mutation carriers who underwent RRBSO and were followed for an average of 7.26 years. Results indicated that among women who were 45 years or younger at the time of their RRBSO, HRT did not increase the odds of breast cancer (OR 0.8 [95% CI 0.3, 1.9]). However, women who were older than 45 years at the time of their RRBSO and used HRT were at significantly higher odds of breast cancer (OR 3.43 [95% CI 1.2, 9.8]). No significant differences in breast cancer rates were observed depending on HRT formulation, with the majority of HRT users receiving EPT [67].

Finally, two additional retrospective studies reported data on HRT use following RRBSO and associated breast cancer cases, even though this association was not their main aim. Gabriel et al. [68] examined 73 *BRCA* mutation carriers and reported that 17.6% of women who used ET and 31% women who did not use HRT developed breast cancer. Similarly, Perri et al. [69] examined 127 matched pairs with *BRCA* mutations and found that among all breast cancer cases, 20% used HRT and 28% did not (*p* = 0.178). The hormone status of the other participants was unclear [69]. Together these studies suggest that HRT use was not associated with more cases of breast cancer [67,68].

Despite the lack of randomized clinical trial evidence, there is considerable high quality observation data that suggests that short-term HRT use of between 3-5 years in *BRCA* mutation carriers following RRBSO-induced premature surgical menopause does not increase the risk of breast cancer. While one study observed an increased risk associated with EPT use among *BRCA1* mutation carriers, this finding has yet to be confirmed, and thus warrants further investigation. In addition, further research is needed to assess the safety of HRT use for longer than 5 years in the *BRCA* population.

**Table 1:** HRT use and risk of breast cancer in *BRCA* mutation carriers.

Study	Design/Follow-up	Sample Size (*n*)	HRT Type and Duration	Breast Cancer (BC) Outcome
Rebbeck et al. [62]	Prospective cohort Mean follow-up: 3.6 years	Total *n* = 462 *BRCA* carriers RRBSO *n* = 155 Mean age at RRBSO: 42.7 years No RRBSO *n* = 307	Estrogen therapy (ET) *n* = 93 Estrogen and progesterone combination therapy (EPT) *n* = 62	RRBSO was associated with reduced breast cancer (BC) risk (HR 0.40 [95% CI 0.18, 0.92]) compared to *BRCA* carriers without RRBSO or HRT use HRT use or any formulation did not influence the BC risk reduction (HR 0.37 [95% CI 0.14, 0.96])
Eisen et al. [64]	Matched case- control	Total *n* = 473 postmenopausal *BRCA1* carriers	ET *n* = 28 BC cases and 40 controls EPT *n* = 19 BC cases and 28 controls Mean duration: BC cases: 4 years Controls: 3.7 years	Decreased odds of BC risk associated with HRT use (OR 0.58 [95% CI 0.35, 0.96]) ET was associated with a statistically significant decrease in BC odds (OR 0.51 [95% CI 0.27, 0.98]) EPT was associated with a nonsignificant decrease in odds of BC (OR 0.66 [95% CI 0.34, 1.27])
Gabriel et al. [68]	Retrospective cohort	RRBSO *n* = 73 *BRCA* carriers Median age at RRBSO: 42 years	HRT users *n* = 33 (45%) ET *n* = 17 (52%) EPT *n* = 14 (42%) Median duration: 2.79 years	ET users: 17.6% developed BC EPT users: 31% developed BC HRT use was not associated with more cases of BC
Domchek et al. [63]	Prospective cohort (extension and follow-up from Rebbeck et al., 2005) Mean follow-up: 5.4 years	Total *n* = 1229 BRCA carriers Mean age at RRBSO among HRT users: 40.8 years Mean age at RRBSO among non-HRT users: 45 years	HRT users *n* = 255 (21%)	HRT use following RRBSO was not associated with an increase in risk of BC in BRCA1 carriers (HR 0.52 [95% CI 0.30, 0.92]) and BRCA2 carriers (HR 0.24 [95% CI 0.05, 1.03]) BC risk was not increased for both ET and EPT
Kotsopoulos et al. [65]	Matched case-control (extension from Eisen et al., 2008)	Total *n* = 864 BRCA1 carriers Pairs of matched BC cases and controls: 432	Total HRT users: Cases *n* = 91 (21%) Controls *n* = 80 (19%) ET users: Cases *n* = 46 Controls *n* = 42 EPT users: Cases *n* = 28 Controls *n* = 41 Mean duration: Cases: 4.42 years Controls: 4.27 years	HRT use was not associated with BC (OR 0.80 [95% CI 0.55, 1.16]) ET and EPT were both not associated with increased or decreased odds of BC. No observed difference between natural menopause (OR 0.72 [95% CI 0.44, 1.18]) and premature surgical menopause (OR 1.06 [95% CI 0.58, 1.96])
Kotsopoulos et al. [66]	Prospective cohort Mean follow-up: 7.6 years	RRBSO *n* = 872 BRCA1 carriers Mean age at surgery: 43.4 years	HRT *n* = 377 (43%) ET *n* = 259 (69%) EPT *n* = 66 (18%) Mean duration: 3.9 years	HRT use after RRBSO was not associated with an increased risk of BC (HR 0.97 [95% CI 0.62, 1.52]) After 10 years of follow-up, the cumulative incidence of BC was significantly lower among ET users compared to EPT users (12% vs 22%, *p* = 0.04) Each year of ET use led to an 8% reduction in BC risk (HR 0.92 [95% CI 0.83, 1.01]) Each year of EPT use led to a nonsignificant 8% increase in risk (HR 1.08 [95% CI 0.92, 1.27])
Michaelson-Cohen et al. [67]	Retrospective cohort Mean follow-up: 7.26 years	RRBSO *n* = 306 Median age at RRBSO: 44 years	HRT *n* = 150 (49%) ET *n* = 26 (17%) EPT *n* = 82 (55%) Median duration: 4 years	RRBSO < 45 years: HRT did not increase the odds of BC (OR 0.8 [95% CI 0.3, 1.9]) RRBSO > 45 years: HRT use was associated with a significantly higher odds of BC (OR 3.4 [95% CI 1.2, 9.8])
Perri et al. [69]	Retrospective cohort Mean follow-up: 8.7 years	Total *n* = 254 BRCA carriers RRBSO vs. no RRBSO matched pairs *n* = 127 Mean age of RRBSO: 42 years	HRT *n* = 62 (24.4%)	Among all BC cases, 20% used HRT and 28% did not (*p* = 0.178) Findings suggest that HRT use was not associated with more cases of BC

Abbreviations: BC, breast cancer; HR, hazard ratio; OR, odds ratio; HRT, hormone replacement therapy; ET, estrogen therapy; EPT, estrogen and progestogen combination therapy; RRBSO, risk reducing bilateral salpingo-oophorectomy.

## 5. Adverse Outcomes following Premature Surgical Menopause among *BRCA* Mutation Carriers

For health care professionals to properly counsel *BRCA* carriers on the long-term health effects of RRBSO and the advantages of HRT use in managing these health risks, more research is needed. The current literature is limited; however, it does suggest that RRBSO-induced surgical menopause in *BRCA* mutation carriers is associated with declines in the quality of life, sexual functioning, cardiovascular outcomes, and bone health (Table 2).

### 5.1. Quality of Life

Finch et al. [58] had 114 *BRCA* mutation carriers complete questionnaires before and one year after RRBSO. Women who were premenopausal at time of surgery (*n* = 75) experienced a significant increase in vasomotor symptoms, including hot flashes, night sweats and sweating, as well as decreased sexual functioning, including altered desire, pleasure, discomfort, and habit. They also reported that HRT use mitigated vasomotor symptoms and decreased vaginal dryness and dyspareunia [58]. These findings are consistent with a more recent study by Hall et al. [70] which followed 140 *BRCA* mutation carriers for an average of 3.5 years. However, despite increased vasomotor symptoms and a decline in sexual functioning, authors reported that premenopausal RRBSO (*n* = 47) did not impact the overall quality of life. HRT use improved some, but not all adverse effects [70]. Another study assessed the psychosocial functioning of *BRCA* carriers (*n* = 62) and found a significant decrease in levels of cancer-related anxiety along with a significant increase in the severity of vasomotor and depressive symptoms following RRBSO [71].

Contrastingly, Chae et al. [72] examined 52 BRCA mutation carriers, 16 (53%) of whom underwent premenopausal RRBSO and 14 (47%) of whom underwent postmenopausal RRBSO. No differences were observed in the mental quality of life, psychosocial status, sexual function, and menopausal symptoms in women with RRBSO compared to no RRBSO. RRBSO was only found to negatively affect the physical quality of life [72].

### 5.2. Cardiovascular Health

Substantial evidence shows that premature surgical menopause has adverse effects on cardiovascular health in the general population; however, limited studies have specifically examined the high-risk *BRCA* population. In 2012, Cohen et al. [56] conducted a retrospective chart review of 226 *BRCA* mutation carriers who had RRBSO. They reported no significant differences in the frequency of hypertension, hyperlipidemia, diabetes, myocardial infarction, or coronary artery disease between women who had RRBSO before the age of 50 (*n* = 144) and those who had RRBSO at age 50 or older (*n* = 82) [56]. In contrast, Powell et al. [75] later reported a lower predicted 10-year risk of CVD among the women who underwent RRBSO under the age of 50 (*n* = 108) compared to those who had RRBSO at the age of 50 or older (*n* = 106). However, given the clear relationship between advancing age and risk for CVD, the roughly 15-year age difference between the premenopausal and post-menopausal comparison groups means that these results should be interpreted with caution, as there is important confounding by age [55,72].

A 2021 cross-sectional study by van Bommel et al. [75] examined the relationship between time since RRBSO with signs of sub-clinical atherosclerosis in 165 *BRCA* mutation carriers. All *BRCA* carriers had undergone RRBSO before the age of 45 and had a minimum of 5 years since surgery. Authors reported that after adjusting for age and other relevant cardiovascular (CVD) risk factors, and after excluding those who used HRT, time since RRBSO was not associated with subclinical atherosclerosis (i.e., carotid intima-media thickness and pulse wave velocity); however, this finding lacked an appropriate control group for comparison. Moreover, cardiovascular risk factors were compared between *BRCA* mutation carriers and age-matched controls from the general population. They found that *BRCA* mutation carriers had lower systolic and diastolic blood pressure, as well as less abdominal obesity and metabolic syndrome at ages 50-59 years, suggesting an improved CVD risk profile [78]. Similarly, a previous study found a more favourable CHD risk profile among women at high risk for ovarian cancer, including but not limited to *BRCA* mutations, compared to the general population [74]. However, authors of these studies suggested that the observed healthier CVD and CHD risk profiles among *BRCA* carriers may be attributed to the self-selection of healthier women seeking RRBSO, beneficial lifestyle changes post-surgery, and survival bias.

A recent population-based retrospective cohort study, conducted by many of the authors of this manuscript, compared the risk of CVD after premenopausal RRBSO among 360 *BRCA* mutation carriers and two age-matched control groups: 1) women who underwent BO for benign conditions (*n* = 3600): and 2) women with intact ovaries who had a hysterectomy or salpingectomy (*n* = 3600) [59]. No difference in CVD risk was observed in *BRCA* carriers compared to women who underwent BO; however, *BRCA* mutations carriers had a significantly higher risk of CVD compared to controls with intact ovaries. Importantly, despite this increase in CVD events, *BRCA* mutation carriers were less likely to be diagnosed with predisposing conditions and less likely to fill cardioprotective medications. Therefore, these findings highlight that the prevention of adverse CVD outcomes can be improved in the *BRCA* population by placing more focus on post-RRBSO follow-up care [59].

Finally, three additional studies have explored CVD and metabolic risks following RRBSO in high-risk women, including but not exclusive to *BRCA* carriers. One case-control study reported that RRBSO was significantly associated with metabolic syndrome (i.e., a group of conditions that co-occur and increase a person’s risk of heart disease, stroke, and type 2 diabetes) compared to the general population [73]. Another study found that 10-year cardiovascular risk estimates were similar in women after RRBSO compared with age-matched women from the general population; however, this study was cross-sectional [76]. Further, upon assessing the impact of HRT, HRT users were found to have lower total cholesterol and waist circumferences. This finding is consistent with a prospective cohort study exploring cardiometabolic risks 12 months following premenopausal RRBSO (*n* = 95). They found that waist and hip ratios significantly increased following RRBSO, however, this increase was prevented by HRT use [77].

The evidence to date suggests that the population of people undergoing premenopausal RRBSO is generally healthier, of higher socioeconomic status (SES), and less predisposed to CVD than the general population [58,73,74,78]. Thus, while the evidence does not clearly indicate a large and significantly increased risk for CVD following premenopausal RRBSO among *BRCA* mutation carriers, given the suggestion that this group has a more favourable CVD profile, the findings from the larger studies with a prolonged follow-up that suggest an increased risk of CVD events are concerning. Future research should investigate the role that HRT plays in reducing CVD risk in this population.

### 5.3. Bone Health

Six studies to date have explored the impact of RRBSO on long-term bone health, specifically in *BRCA* mutation carriers. Garcia et al. [81] examined the management of osteoporosis in 225 *BRCA* carriers following RRBSO. Over a median follow-up of 41 months, they reported that 4.4% of *BRCA* carriers had a fracture after surgery. Further, of the 44% (*n* = 99) of women who had at least one dual-energy X-ray absorptiometry (DEXA) scan, 56% had results indicating osteopenia and 12% had results consistent with osteoporosis. Compared to the general population, the incidence of osteoporosis was higher among those post-RRBSO who had a DEXA scan (5.3% vs. 12%, respectively); however, it was not significantly different compared to the 7% osteoporosis rate in the entire cohort. When interpreting these results, it is important to note that the average age at RRBSO was 50 years, and no information was provided about the menopausal status at RRBSO. In addition, only 25% (*n* = 14) of women who had RRBSO used HRT, and due to such a low number of women using HRT and receiving DEXA scans, authors were unable to assess the impact of HRT use on DEXA results. Overall, a key observation of this study was that despite the increased risk of bone loss following RRBSO, the majority of women undergoing RRBSO are not being screened routinely with DEXA scans [81].

Powell et al. [82] reported an elevated prevalence of bone loss among *BRCA* mutation carriers following RRBSO. Of the 238 women included in this prospectively collected cohort, the prevalence of bone loss was 72.5% among women with RRBSO compared to 55% among women with intact ovaries. Additionally, 6.9% of BRCA mutation carriers had a fracture following RRBSO. Comparing those who had their RRBSO at premenopausal ages with those who had a postmenopausal RRBSO, there was no significant difference in the fracture rates. With regard to bone loss, osteopenia and osteoporosis were more frequent in women who underwent postmenopausal RRBSO compared to premenopausal RRBSO; however, the 12-year gap between the average ages of these two comparison groups makes them incomparable with respect to these highly age-dependent outcomes [82].

Another study compared DEXA scans of 152 *BRCA* mutation carriers. Cohen et al. [56] reported that 70% of women who underwent RRBSO below the age of 50 and 72% of women who underwent RRBSO above the age of 50 were found to have osteopenia or osteoporosis as seen on DEXA scans. Despite the large mean age difference between these two comparison groups (premenopausal-RRBSO: 44.7 years vs. postmenopausal-RRBSO: 60.6 years), their bone health assessments were equivalent, suggesting the premenopausal women undergoing RRBSO had significant losses in bone density. In contrast, an observational study by Chapman et al. [79] examined osteopenia and osteoporosis diagnoses after RRBSO in 51 *BRCA* mutation carriers. After a median of 6 years follow-up, there were 31 (61%) DEXA scan results available, and none of the three (10%) osteoporosis cases occurred among women who underwent RRBSO before the average age of natural menopause. The small sample size of this study limits conclusions.

In 2019, Kotsopoulos et al. [79] conducted a longitudinal retrospective cohort study to compare pre- and post-RRBSO DEXA scans of 95 *BRCA* mutation carriers. After a mean follow-up period of 22 months, BMD loss was observed in the 50 women who were premenopausal and the 45 women who were postmenopausal at the time of surgery. In addition, there was a significantly greater annual decrease in BMD among women who were premenopausal at RRBSO compared to women who were postmenopausal at RRBSO. Authors also reported that HRT use after premenopausal RRBSO was associated with reduced bone loss compared to non-users (-2.00% vs -4.69%, *p* = 0.02 for lumbar spine; and -1.38% vs. -3.21%, *p* = 0.04 for total hip) [83].

Most recently, Abreu do Valle et al. [60] conducted a population-based retrospective cohort study to investigate the risk of osteoporosis and fractures among women with *BRCA* mutations who underwent RRBSO before the age of 50 (*n* = 329). Results were compared with two age-matched groups without known mutations: 1) women without *BRCA* mutations who underwent BO (*n* = 3290); and 2) women without *BRCA* mutations with intact ovaries who had hysterectomy or salpingectomy (*n* = 3290). After a median follow-up time of 6.9 years, there was a higher risk of osteoporosis (aHR 1.60 [95% CI 1.00, 2.54] compared to women who had BO; and aHR 2.49 [95% CI 1.44, 4.28] compared to women with intact ovaries). No increased fractures were observed for *BRCA* mutation carriers; however, conclusions were limited by the young age of the study cohort, and the authors noted that with further follow-up, the expected differences in fracture rates would emerge. A decreased risk of receiving an osteoporosis diagnosis was also observed among women with *BRCA* mutations who used HRT. This finding is consistent with that of Kotsopoulos et al. and suggested that HRT is maintaining bone density in this population, following premenopausal RRBSO. In addition, only 46% of *BRCA* mutation carriers received DEXA scans following RRBSO, and of those diagnosed with osteoporosis, only 36% received bisphosphonates. Together these results highlight that bone health promotion can be improved in this population with focused post-surgical care and bone protection [60].

Two additional studies in women at high risk for ovarian cancer (including but not limited to *BRCA* mutation carriers) assessed the impact of HRT use on bone health following RRBSO. A retrospective study from Challberg et al. [80] found that the prevalence of reduced bone mass was much greater among women who had over 24 months of estrogen deprivation compared to those who used HRT [80]. Further, a 2020 prospective study found that premenopausal RRBSO was associated with a substantial loss of bone density and bone strength, and that HRT use appeared to mitigate the loss of bone density and bone stiffness [84].

Taken together, these studies point to an important adverse effect of premenopausal RRBSO among those with *BRCA* mutations on bone density, the risk of osteopenia, and the risk of osteoporosis. While studies are limited by small sample sizes and young mean ages of cohorts, it is probable that these differences will translate into differences in fracture risk with extended follow-up and larger sample sizes. There is also evidence indicating that HRT is important in reducing the bone density losses in people who underwent premenopausal RRBSO, and thus the improved use of HRT in this population could mitigate some of this risk.

## 6. Conclusions

In summary, RRBSO remains the gold standard preventative option for *BRCA* carriers at high risk for ovarian and breast cancers. However, when performed at the recommended ages, RRBSO induces immediate premature surgical menopause, along with the accompanying adverse psychosocial, cardiovascular, and bone health consequences. HRT use until the average age of natural menopausal can help mitigate these health risks, yet current data show that HRT uptake is low and sustained use is not long enough. We believe that the substandard HRT use is at least partially a result of well-intentioned concerns around the role that HRT might play in increasing breast cancer risk in this population that is already at dramatically increased risk for breast cancer. However, we now have a large amount of high-quality observational research (randomized controlled trial evidence will not be generated on this topic, given the lack of clinical equipoise) that strongly points to no increased breast cancer risk in this population following up to 5 years of HRT use. Thus, the evidence strongly supports the short-term safety of HRT use among *BRCA* carriers and further research is needed to confirm the safety of long-term HRT use in this population.

While there is currently no evidence that HRT use increases breast cancer risk among *BRCA* carriers following premenopausal RRBSO, there is growing evidence of adverse outcomes in non-cancer endpoints. The adverse outcomes appear to be at least partially mitigated by HRT, for example, bone density loss. Thus, we recommend concerted efforts to improve the uptake and prolonged use of HRT in this population. We would be remiss if we did not also remind readers of the important quality of life issues at play. Women with *BRCA* mutations who enter premature surgical menopause and initiate and maintain an appropriate HRT regimen report a higher quality of life, better sexual health, improved sleep, and reduced depressive symptoms [57,69]. Thus, we believe that the evidence supports activities that will spread awareness that HRT use post-surgical menopause is safe among *BRCA* mutation carriers, that it does not counteract the breast cancer risk reduction of undergoing RRBSO, and is important in the *BRCA* population for both maintaining quality of life and reducing long-term adverse outcomes.

## Figures and Tables

**Table 2:** Long-term non-cancer risks in people with *BRCA* mutations following risk-reducing bilateral salpingo-oophorectomy.

Study	Design/Follow-up	Sample Size (*n*)/ Age (years)	Health Outcomes	Comparison	Results
Quality of Life					
Finch et al. [58]	Prospective cohort Mean follow-up: 13.6 months	Total *n* = 114 BRCA carriers Premenopausal RRBSO *n* = 75 Mean age at RRBSO: 44.7 Postmenopausal RRBSO *n* = 39 Mean age at RRBSO: 52.7	Vasomotor and physical symptoms, sexual and psychosocial functioning	Premenopausal vs. postmenopausal RRBSO HRT users vs. non-users	Premenopausal RRBSO was associated with significantly worse vasomotor symptoms and decreased sexual functioning HRT use: significantly fewer vasomotor symptoms and improved sexual functioning than no HRT use
Hall et al. [70]	Prospective cohort Mean follow-up: 3.5 years	Total *n* = 140 BRCA carriers Premenopausal RRBSO *n* = 93 Mean age at RRBSO: 43.8 years Postmenopausal RRBSO *n* = 47 Mean age at RRBSO: 52.4 years	Vasomotor and physical symptoms, sexual and psychosocial functioning, and quality of life (QoL)	Premenopausal vs. postmenopausal RRBSO HRT users vs. non-users	Premenopausal RRBSO was associated with increased vasomotor and physical menopausal symptoms and decreased sexual function, with no impact on QoL HRT users had fewer symptoms than non-users, yet HRT did not eliminate all negative effects
Stanisz et al. [71]	Prospective cohort Mean follow-up: 353 days	Total *n* = 61 BRCA carriers Mean age: 44.7 years	QoL and psychosocial functioning	Before RRBSO vs after RRBSO	QoL after RRBSO: significant decline in domains of somatic and vasomotor symptoms, memory/concentration, and sexual and sleep behaviours Psychosocial functioning after RRBSO: significant decrease in level of anxiety and a significant increase in severity of climacteric and depressive symptoms
Chae et al. [72]	Cross-sectional	Total *n* = 52 BRCA carriers RRBSO *n* = 30 Mean age at RRBSO: 49.8 years Premenopausal RRBSO *n* = 16 Postmenopausal RRBSO *n* = 14 years No RRBSO *n* = 22 Mean age: 42.1 years	QoL, anxiety, depression, optimism, sexual function, and menopausal symptoms	RRBSO vs no RRBSO Premenopausal vs. postmenopausal RRBSO	RRBSO uptake was associated with worse physical QoL No significant differences in mental QoL, psychosocial status, sexual function, and menopause symptoms from undergoing RRBSO Mental QoL was significantly lower in postmenopausal RRBSO
Cardiovascular Health					
Michelsen et al. [73]	Case-control Mean follow-up: 6.5 years	Cases: RRBSO *n* = 326 BRCA carriers or those at increased hereditary risk Controls: No RRBSO *n* = 679 general population with BRCA status unknown	Metabolic syndrome	RRBSO vs. no RRBSO	RRBSO was significantly associated with metabolic syndrome according to the 2005 National Cholesterol Education Program Adults Treatment Panel III criteria and the International Diabetes Federation criteria
Michelsen et al. [74]	Case-control Mean follow-up: 6.5 years	Cases: RRBSO *n* = 326 BRCA carriers or those at increased hereditary risk Controls: No RRBSO *n* = 1630 general population with BRCA status unknown	Coronary heart disease (CHD)	RRBSO vs. no RRBSO	Compared to controls, RRBSO cases had an overall more favorable CHD risk profile, including: more physical activity, lower levels of total cholesterol, higher levels of high-density lipoprotein cholesterol, lower systolic blood pressure, and lower body mass index (BMI) compared to controls RRBSO was associated with a lower mean Framingham 10-year risk score
Cohen et al. [56]	Cross-sectional Mean time since RRBSO: 8.4 years	Total *n* = 226 BRCA carriers RRBSO < 50 *n* = 144 Mean age: 44.3 RRBSO ≥ 50 *n* = 82 Mean age: 60.1	Hypertension, diabetes mellitus, hypercholesterolemia, coronary artery disease (CAD) or myocardial infarction (MI)	RRBSO < 50 years vs. RRBSO ≥ 50 years	RRBSO < 50 years: Hypertension: 13% Diabetes mellitus: 0.7% Hypercholesterolemia: 15% CAD or MI: 1.4% RRBSO ≥ 50 years: Hypertension: 21% Diabetes mellitus: 4% Hypercholesterolemia: 18% CAD or MI: 4% No significant differences between RRBSO < 50 and RRBSO ≥ 50 groups
Powell et al. [75]	Cross-sectional RRBSO < 50 Median time since RRBSO: 8 years RRBSO ≥ 50 Median time since RRBSO: 6.5 years	Total *n* = 233 RRBSO < 50 years *n* = 108 Median age: 51 RRBSO ≥ 50 years *n* = 106 Median age: 63.5 No RRBSO *n* = 19 Median age: 56	Hypertension, diabetes mellitus, hyperlipidemia, stroke, MI, cardiac surgery, atherosclerotic cardiovascular disease (ASCVD)	RRBSO < 50 years vs. RRBSO ≥ 50 years	RRBSO < 50 years: Hypertension: 21.3% Diabetes mellitus: 6.5% Hyperlipidemia: 25% Stroke: 3.7% MI: 2.8% Cardiac surgery: 0.9% RRBSO ≥ 50 years: Hypertension: 34.0% Diabetes mellitus: 10.4% Hyperlipidemia: 32% Stroke: 3.8% MI: 2.8% Cardiac surgery: 0 No significant differences in CVD outcomes between RRBSO < 50 and RRBSO ≥ 50 years RRBSO ≥ 50 had a higher 10-year risk of ASCVD
Johansen et al. [76]	Retrospective cohort Mean follow-up: 4.2 years	RRBSO *n* = 134 Mean age: 47 years No RRBSO *n* = 268 Mean age: 46 years	Cardiovascular disease (CVD), cardiometabolic factors	RRBSO vs. no RRBSO HRT users vs. non-users	10-year CVD risk estimates were similar in those with RRBSO compared to age-matched controls RRBSO group had lower BMI and waist circumference HRT users had lower total cholesterol and waist circumferences, but comparable CVD risk to non-users
Abreu do Valle et al. [59]	Retrospective cohor tMean follow-up: RRBSO: 6.3 years Bilateral oophorectomy (BO): 9.8 years Intact ovaries: 8.9 years	RRBSO *n* = 360 Mean age: 42.5 years BO without BRCA mutation *n* = 3600 Mean age: 42.6 years Intact ovaries without BRCA mutation *n* = 3600 Mean age: 42.6 years	CVD, predisposing conditions, use of cardioprotective medications	Premenopausal RRBSO vs. BO Premenopausal RRBSO vs. hysterectomy or salpingectomy with intact ovaries	No significant increased risk for CVD between RRBSO and BO groups, but the RRBSO group was less likely to be diagnosed with predisposing conditions Compared to women with intact ovaries, RRBSO group was associated with a significant increased CVD risk and was less likely to be diagnosed with predisposing conditions or to fill cardioprotective medications
Hickey et al. [77]	Prospective cohort Mean follow-up: 12 months	RRBSO *n* = 95 (women at high risk for ovarian cancer, not limited to BRCA carriers) Mean age at baseline: 42.1 years Intact ovaries *n* = 99 (unknown BRCA status) Mean age at baseline: 40.8 years	Cardiometabolic risk factors	Premenopausal RRBSO vs no RRBSO HRT user vs non-user	Blood pressure and circulating cardiometabolic risk factors were overall unchanged at 12 months post-RRBSO. Increased BMI, weight, waist circumference, and waist-hip ratio after RRBSO compared to controls. These increases were non-significant after adjusting for baseline values. HRT users had a significantly lower mean waist circumference compared to non-HRT users
van Bommel et al. [78]	Cross-sectional Median time since RRBSO: 9.5 years	Total *n* = 165 BRCA carriers Median age: 49 years	Signs of sub-clinical atherosclerosis: carotid intima-media thickness (CIMT) and pulse wave velocity (PWV)	RRBSO vs. no RRBSO (general population)	CIMT: 692.7 μm PWV: 6.40 m/s Time since RRBSO was not associated with subclinical atherosclerosis as measured by CIMT and PWV Compared to a reference group from the general population, BRCA carriers with RRBSO were similar with regard to BMI, diabetes, total cholesterol, high-density lipoprotein cholesterol, and smoking, but had lower systolic and diastolic blood pressure
Bone Health					
Chapman et al. [79]	Cross-sectional Median time since RRBSO: 6 years	Total *n* = 51 BRCA carriers Median age at RRBSO: 46	DEXA scans, osteoporosis, osteopenia	No comparison group	DEXA scan: 75% Osteopenia: 23% Osteoporosis: 10%
Challberg et al. [80]	Retrospective cohort	Total *n* = 212 BRCA carriers or those at increased hereditary risk who underwent premenopausal RRBSO Mean age at RRBSO: 41 years	DEXA scans, osteoporosis, osteopenia	HRT users vs. non-users	DEXA scan: 56% Osteopenia: 28% Osteoporosis: 10% HRT use (current and not current): 63% The prevalence of reduced bone mass was far higher among women who had > 24 months of estrogen deprivation than HRT users
Cohen et al. [56]	Cross-sectional Mean time since RRBSO: 8 years DEXA scan done a mean of 3.2 years after RRBSO	Total *n* = 152 BRCA carriers with DEXA scans RRBSO < 50 years *n* = 80 Mean age at RRBSO: 42.9 RRBSO ≥ 50 years *n* = 64	Osteoporosis, osteopenia	RRBSO < 50 years vs. RRBSO ≥ 50 years	RRBSO < 50 years: Osteopenia: 61% Osteoporosis: 9% RRBSO ≥ 50 years: Osteopenia: 52% Osteoporosis: 20% No significant differences in abnormal DEXA scans between the two comparison groups
Garcia et al. [81]	Retrospective cohort Median follow-up: 41 months	Total *n* = 225 BRCA carriers Mean age at RRBSO: 50	DEXA scans, osteoporosis, osteopenia, fractures	No comparison group	DEXA-scan: 44% Osteopenia: 55.6% Osteoporosis: 12.1% Fractures: 4.4% Osteoporosis in women with DEXA scan results was higher than US national prevalence
Powell et al. [82]	Cross-sectional	Total *n* = 238 BRCA carriers Premenopausal RRBSO *n* = 112 Median age at RRBSO: 45 Postmenopausal RRBSO *n* = 106 Median age at RRBSO: 57 No RRBSO *n* = 20	Bone loss, osteoporosis, osteopenia, fractures	RRBSO vs. no RRBSO Premenopausal vs. postmenopausal RRBSO	No RRBSO: Bone loss: 55% Osteoporosis: 5% Fractures: N/A Premenopausal RRBSO: Bone loss: 63.4% Osteoporosis: 11.6% Fractures: 5.4% Postmenopausal RRBSO: Bone loss: 82.1% Osteoporosis: 16% Fractures: 8.5% No significant differences in bone loss between RRBSO and no RRBSO Lower bone loss for premenopausal RRBSO, but no significant differences in osteoporosis and fractures between premenopausal and postmenopausal RRBSO groups
Kotsopoulos et al. [83]	Retrospective cohort Mean follow-up: 22 months	Total *n* = 95 BRCA carriers Premenopausal RRBSO *n* = 50 Mean age at RRBSO: 40 Postmenopausal RRBSO *n* = 45 Mean age at RRBSO: 52.4	Change in bone mineral density before and after RRBSO	Premenopausal vs. postmenopausal RRBSO HRT users vs. non-users	Premenopausal RRBSO: Lumbar spine: -3.45% Femoral neck: -2.85% Total hip: - 2.24% Postmenopausal RRBSO: Lumbar spine: -0.82% Femoral neck: -0.68% Total hip: -0.18% (not significant) Greater annual decrease in BMD in premenopausal RRBSO HRT was associated with less annual change in BMD
Jiang et al. [84]	Prospective cohort Mean follow-up: 24 months	Premenopausal RRBSO *n* = 30 (women at high risk for ovarian cancer, not limited to BRCA carriers) Mean age: 42.6 years Intact ovaries *n* = 42 (unknown BRCA status) Mean age: 40.2 years	DEXA scans, areal bone mineral density (aBMD), bone strength	Premenopausal RRBSO vs. no RRBSO HRT users vs. non-users	aBMD at lumbar spine: -4.7% Tibial volumetric cortical density: -1.0% Tibial bending stiffness: -12.1% RRBSO resulted in a significant loss of bone density and bone strength at 24 months compared to baseline HT prevented loss of bone density and bone stiffness, although there was still a modest decrease in lumbar spine aBMD in HT users.
Abreu do Valle et al. [60]	Retrospective cohort	RRBSO *n* = 329 Mean age: 42.4 years BO without BRCA mutation *n* = 3290 Mean age: 42.5 years Intact ovaries without BRCA mutation *n* = 3290 Mean age: 42.5 years	Risk of osteoporosis and fractures, DEXA scans, bisphosphonates use	Premenopausal RRBSO vs. BO Premenopausal RRBSO vs. hysterectomy or salpingectomy with intact ovaries HRT users vs. non-users	There was no increased risk of fractures associated with RRBSO compared to BO and intact ovaries Among those with available DEXA scans, RRBSO was associated with a higher risk of osteoporosis compared to BO and intact ovaries 36% of women with osteoporosis post-RRBSO received bisphosphonates HRT users were less likely to be diagnosed with osteoporosis

Abbreviations: RRBSO, risk-reducing salpingo-oophorectomy; BO, bilateral oophorectomy; HRT, hormone replacement therapy; QoL, quality of life; CHD, coronary heart disease; CAD, coronary artery disease; CVD, cardiovascular disease; ASCVD, atherosclerotic CVD; CIMT, carotid intima-media thickness; PWV, pulse wave velocity; MI, myocardial infarction.
